# Testing sampling effort and relative abundance descriptors of belowground ectomycorrhizal fungi in a UK planted scots pine woodland

**DOI:** 10.1080/21501203.2017.1394393

**Published:** 2017-11-15

**Authors:** Luis Villarreal-Ruiz, Cecilia Neri-Luna

**Affiliations:** a Laboratorio de Recursos Genéticos Microbianos y Biotecnología (LARGEMBIO), Postgrado en Recursos Genéticos y Productividad- Genética, Colegio de Postgraduados, Texcoco, México; b Laboratorio de Ecofisiología Vegetal, Departamento de Ecología, CUCBA, Universidad de Guadalajara, Zapopan, México

**Keywords:** UK Caledonian pinewood, *Pinus sylvestris*, ectomycorrhizal fungi, *Meliniomyces bicolor*, *Cortinarius*

## Abstract

The native Caledonian pinewoods of Scotland, UK contain a unique and unexplored biodiversity of below-ground ectomycorrhizal fungi which may constitute a valuable source of microbial genetics resources for woodland restoration. In this study we test the sampling effort, taxa detection (ectomycorrhiza morpho-anatotyping) and relative abundance measurements (ectomycorrhizal root tip number vs. dry weight) of below-ground ectomycorrhizal fungal communities in a planted *Pinus sylvestris* Caledonian woodland. A total of 18 replicated sampling points were set up at differential distance along a 125 m transect, in a 50-yr-old, stem exclusion: thicket phase, *Pinus sylvestris* pinewood stand. A total of 11 ectomycorrhizal morpho-anatotypes were detected from 6689 ectomycorrhizal root tips counted and weighing 992.6 mg dry weight. The major ectomycorrhizal taxa were *Meliniomyces bicolor* and *Cortinarius* spp. accounting ~50% of total pine roots. A highly significant relationship (*r^2^* = 0.16, *p* < 0.000) was found between Sørensen dissimilarity in soil cores and distance apart. In this study, the spatial arrangement of samples indicated that over short distances the dissimilarity was lower in contrast with the longer distances along the transect.

## 1. Introduction

Scots pine (*Pinus sylvestris* L.) is the most widespread European pine and is distributed from southern boreal habitats to Atlantic and central continental Europe (Richardson and Rundel 1998; Tóth et al. 2017). In Scotland, the remnant Caledonian native pine woodland (*Pinus sylvestris* L.) is a species-rich habitat harbouring a characteristic and specialised biota (Quine and Humphrey 2010; Bain 2013). Ectomycorrhizal (EcM) fungi are species-rich communities playing a central role in ecosystem processes in boreal forests and native pinewoods (Dahlberg 2001; Lindea van der et al. 2010; Tedersoo et al. 2014). They establish complex bipartite and tripartite mutualistic networks with overstorey and understorey plants allowing them to share nutrient resources and relaxing the competition (Villarreal-Ruiz et al. 2004, 2012; Fodor 2013). Estimations of fungal species richness in Scotland suggest that 8000–15,000 taxa are present and between 35 and 40% are mycorrhizal, mainly based on above-ground sporocarp presence (Watling 1997; Iskipp 2000). In contrast, the EcM fungi below-ground presence and dynamics is poorly studied in native pine woodlands regardless of its relevance to generate predictive tools to improve management plans to overcome the impact of human activities that restricted their 1.5 million ha original surface to less than 1% (Newton et al. 2001). For this reason, restoration of native Caledonian pinewoods represents a priority for the EU Habitats and Species Directive, the UK’s Biodiversity Action Plan and the Scottish Natural Heritage, the statutory conservation agency, in order to maintain their unique biodiversity and sustainable use in the long term (Hampson and Peterken 1998; Forestry Commission 2003; Featherstone 2010).

This research was undertaken to study the biodiversity and ecology of below-ground ectomycorrhizal fungi in a Scots pine (*P. sylvestris* L.) planted woodland to answer the following questions:

1. What is the relationship between number and dry weight (DW) of ectomycorrhizal fungal morpho-anatotypes as descriptors of relative abundance?
2. Are samples taken close together more similar in species composition and community structure than to those taken further apart?

## 2. Materials and methods

### 2.1. Study area and stand description

This study was performed in a 50-year-old, (stem exclusion: thicket phase, *sensu* Mason et al. 2004) planted (*Pinus sylvestris* L.) Caledonian pinewood (stand 8, 57° 02ʹ 01ʺ N, 002° 52ʹ 52ʺ W, 214 m a.s.l.) as part of an EcM chronosequence pilot study at Glen Tanar Nature Reserve, Aberdeenshire, NE Scotland, UK (National Grid Reference No. 470950, see Villarreal-Ruiz 2006), Figure 1. Because it was difficult to locate a 50-year-old naturally regenerated stand in order to construct the full chronosequence, the problem was overcome by selecting a large plantation within the forest area that met most of Oliver’s (1982) selection criteria. Scots pine seedlings were planted from local seeds and some naturally regenerated seedlings might also be present (Callander and Mac Kenzie 1991; Atterson and Ross 2002). The study site is part of the Grampian Region and the climate is of the cool temperate oceanic type (Steven and Carlisle 1959; Clark 1995). The soil type in Glen Tanar is predominantly freely drained podzols derived from granitic solifluction deposits with a thin layer of raw humus (FitzPatrick 1977; Vickers and Palmer 2000). The overstorey vegetation is dominated by *Pinus sylvestris* L., and the understorey plants are *Calluna vulgaris* L. (Hull.), *Vaccinium myrtillus* L, *V. vitis-idaea* L. and *Deschampsia flexuosa* (L.) Trim.

**Figure 1.**
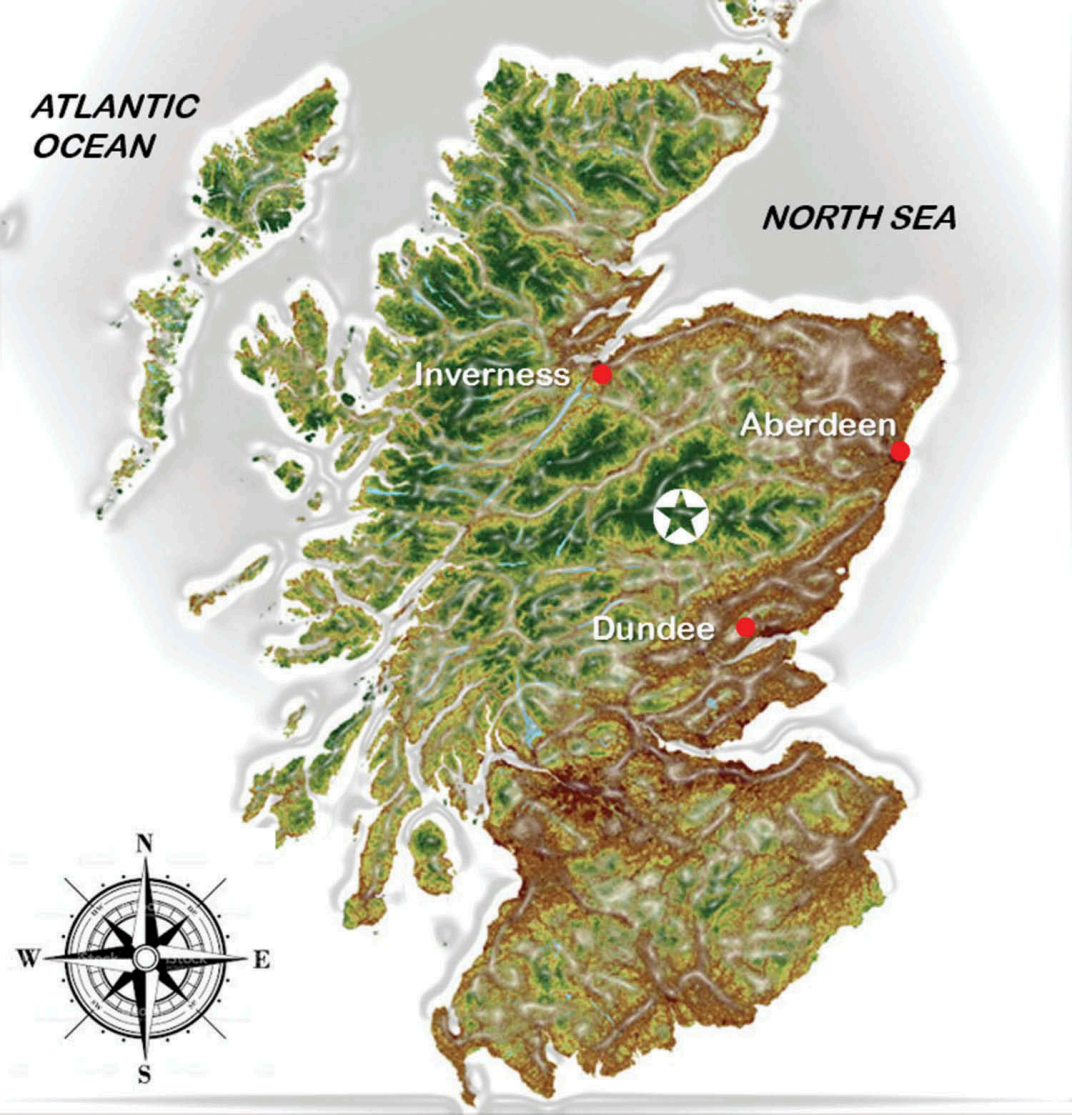
Study area geographic location (✪) of Glen Tanar National Nature Reserve with Caledonian native pine woodland (*Pinus sylvestris* L.), after Anonymous (2014).

### 2.2. Experimental design and samples processing

**Figure 2.**
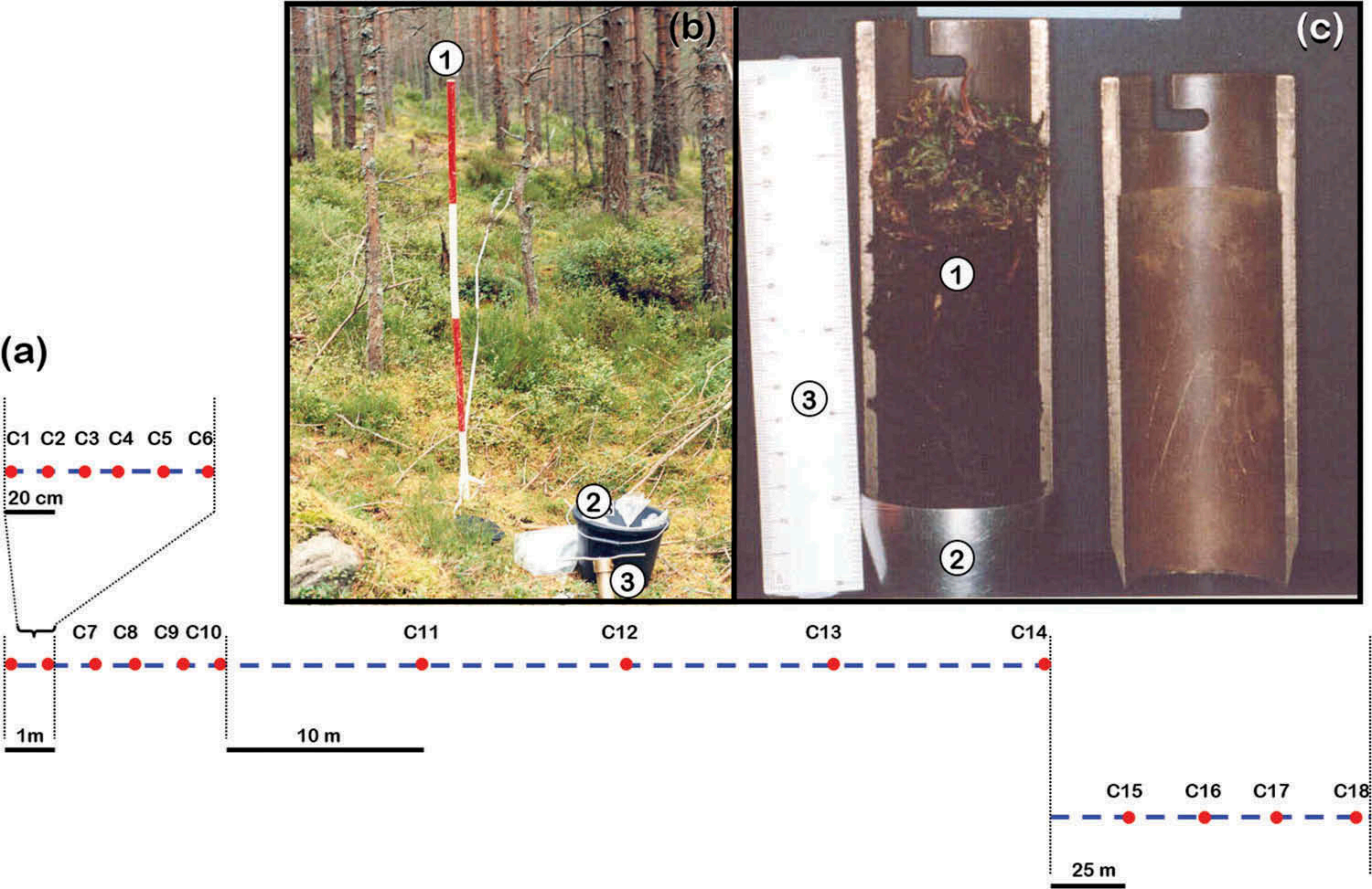
Sampling procedure along a 125 m transect with replicated sampling points at differential set points in a 50-year-old Scots pine (stand 8) at Glen Tanar Nature Reserve. (a) Illustration of transect line (dashed line) and replicated sampling points (dots). (b) Field sampling procedure in stand 8. (1) transect line, (2) soil samples in a bucket, (3) split soil core sampler. (c) Detail of soil sample (1) in the split soil core sampler (2) (3) Ruler 15 cm.

A total of 18 soil cores were taken at set distances along a 125 m transect with replicated sampling points as follows: (1) 6 each 20 cm (2) 4 each 1 m, (3) 4 each 10 m, (4) 4 each 25 m, Figure 2 (a-b). Soil cores were taken with a split soil core sampler (diameter 4.5 cm; depth 12 cm, Figure 2(c). Each time the corer was driven into the soil profile as far as possible and obstructions such as stones and rocks affected the depth sampled were removed. Soil samples were wrapped with aluminium foil, placed in sealed plastic bags and transferred to the lab and kept in a cold room at 4°C until processed. Each soil sample was individually soaked for 30 min in tap water and transferred to a sieve series (0.5, 1.0 and 2 mm).

### 2.3. Data collection and measurements

The living EcM root tips were carefully hand-sorted using fine forceps under a Wild M5 Heerbrugg stereo microscope (12×) by wet sieving. The pine ectomycorrhizal root tips were characterised morphologically (morphotyping) using mantle colour and texture of extramatical hyphae or rhizomorphs, root tip branching pattern, and presence, colour and general morphology and texture of extramatrical hyphae and/or rhizomorphs (Agerer 1991). Each ectomycorrhizal morphotype was photographed in a Leica stereomicroscope and the most abundant ectomycorrhizal types were briefly described following Agerer (2002). In order to corroborate the homogeneity of EcM morphotypes groups, the anatomical characterisation (anatotyping) was performed by free hand longitudinal and cross-sections (in order to confirm the presence of a Hartig net), mantle scrapes and rhizomorphs mounted first in water and later in 85% lactic acid (Agerer 1991). The mounted slides were observed using phase contrast, differential interference contrast and fluorescence microscopy at ×40 to ×100 magnification, and photographed using a Carl Zeiss Axiophot D-7082 photomicroscope. Duplicates of single EcM types were preserved in FEA solution (formaldehyde:ethanol 70%: acetic acid (5: 90: 5 v/v/v) as voucher specimens following Agerer et al. (2000). Sorted homogeneous EcM morpho-anatotypes were counted and dried at 60°C for 48 h and placed in a perspex single door desiccator chamber with silica gel and subsequently the DW of each EcM morpho-anatotype taxa was recorded using a Mettler AJ100 analytical balance. A database of all ectomycorrhizal types detected was constructed based on macro and/or microscopic observations following Agerer (2002), and subsequently identified using classical methods. The exploration type of each EcM morph-anatotype was determined accordingly with Agerer (2001).

### 2.4. Data analysis

The multivariate analysis was performed using different routines in PC-ORD version 3.0 and 4.0 for Windows (McCune and Mefford 1999) as follows: The total number of EcM taxa across all the sample replicated points along the transect was estimated accordingly with McCune and Grace (2002). The “true” species richness in the study area was estimated using: (a) the first-order jackknife estimator (Heltshe & Forrester 1993; Palmer 1990):

$$Jack1=S+r1\;\left(n-1\right)/n$$

where *S* = the observed number of species; *r*1 = the number of species occurring in one sample unit; *n* = the number of sample units.

(b) The second-order jackknife estimator (Burnham and Overton 1979; Palmer 1991):

$$Jack2=S+r1\;\left(2n-3\right)/n-r2{\left(n-2\right)}^{2}/\left(n\left(n-1\right)\right)$$

where *r*2 = number of species occurring in exactly two sample units.

A dissimilarity index between all possible pairs of cores along the transect was computed using routines in PC-ORD. The index used was the Sørensen (Bray and Curtis 1957) ecological distance measure (McCune and Mefford 1999). Statistics were performed by using SPSS® v. 12.0.1. package. EcM fungal parameters: number of EcM root tips versus DW were plotted by regression analyses (Dytham 2003).

## 3. Results

### 3.1. Species richness and frequency

**Figure 3.**
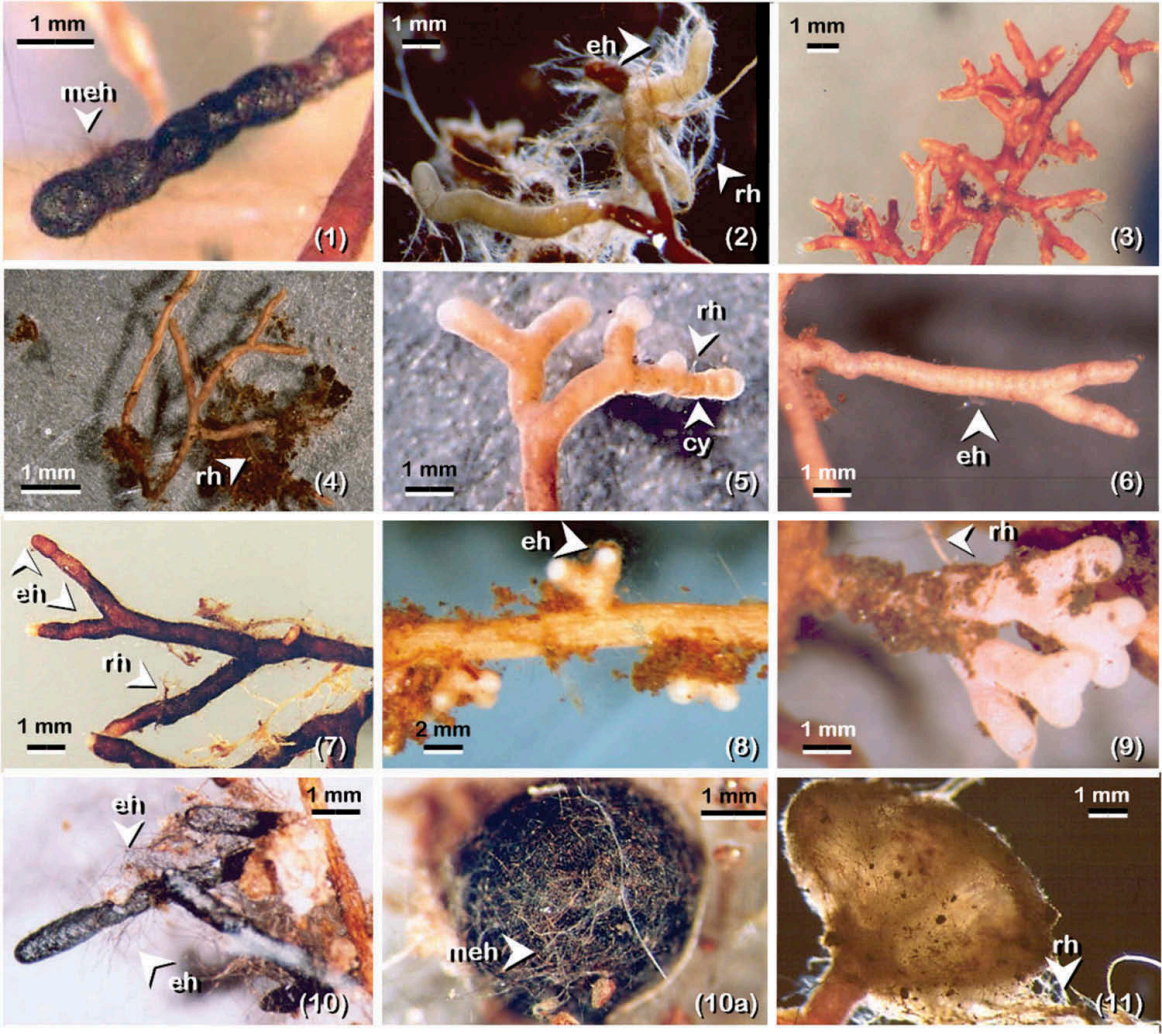
Ectomycorrhizal morpho-anatotypes associated with *Pinus sylvestris* in the experimental stand at Glen Tanar Nature Reserve; the order is based on relative abundance (in percent of number) as in Table 1. (1) *Meliniomyces bicolor* with complete melanised mantle covered with melanised emanating hyphae (meh); (2) *Cortinarius* spp. showing emanating hypae (eh) and rhizomorph (rh); (3) Unknown 1; (4) *Lactarius* sp.1 with rhizomorph (rh); (5) *Gomphidius* cf. *roseus* with distinctive profuse cystidia (cy) on outer mantle and short rhizomorph (rh); (6) *Lactarius* cf. *rufus* showing very fine emanating hypa (eh); (7) *Pseudotomentella tristis* with emanating hypa (eh) and rhizomorph (rh); (8) *Lactarius* sp.2 with amanating hypha (eh); (9) Unknown 2; (10) *Cenococcum geophilum* short distance ET-EcM with emanating hypa (eh); (10a) *Cenococcum geophilum* sclerotia covered with melanised hypae (mh); (11) *Suillus variegatus* long distance ET-EcM with rhizomorph (rh).

A total of 11 EcM morpho-anatotypes fungal groups were recognised (Table 1, Figure 3) from a total of 6689 pine root tips recovered and counted from the 18 soil cores taken along the transect. The total biomass of the EcM morpho-anatotypes fungal community was 992.6 mg DW.

The major EcM fungal taxa were *Meliniomyces bicolor* Hambl. and Sigler and *Cortinarius* spp. colonising around 50% of pine roots, followed by nine taxa colonising the remaining pine roots. It is important to state that all pine roots recovered were colonised by EcM fungi. The five most frequent EcM taxa found in soil cores along the transect were: *Cortinarius* sp. (14/18) followed by *M. bicolor* (13/18), “Unknown” and *Lactarius* sp. (7/18) each, and *Cenococcum geophilum* Fr. (6/18).

**Table 1.** EcM taxa found in the Scots pine plantation based on morpho- and/or anatomy, exploration-types and percentage of overall relative abundance in number.

TF	Taxa	Rh	MT	Cy	Lh	An	Hs	Cl	Co	Sh	ET	%ORAN
1	*Meliniomyces bicolor*	-	C	-	-	S	S-R	-	D’BR-BK	Unr	SD	25.79
2	*Cortinarius* spp.	A	B	-	-	C	S	+	WT	Irr-Pin	MF	24.43
3	Unknown 1	-	A	-	+	-	S	-	OR-YL	Dic-Cor	C	14.96
4	*Lactarius* sp.1	B	B	-	+	S	S	+	YL-OR	Dic	C	13.74
5	*Gomphidius* cf. *roseus*	A	D	A	-	-	S	-	YE-OR-RD	Dic	MS-PB	4.80
6	*Lactarius* cf. *rufus*	-	M	-	+	-	S	-	OR	Dic	C	3.92
7	*Pseudotomentella tristis*	B	A	-	-	O	S	-	BR	Dic	MS	3.80
8	*Lactarius* sp. 2	-	A	-	+	-	S	-	YE-OR	Dic	C	3.69
9	Unknown 2	-	-	-	-	-	-	-	WT-RD	Dic	C	2.75
10	*Cenococcum geophilum*	-	G	-	-	S	S	-	BK	Unr	SD	1.60
11	*Suillus variegatus*	F	A-F	-	-	S	S-R	-	BR	Tub	LD	0.52^a^

TF: ectomycorrhizas taxa key number used in Figure 3. Rh: rhizomorph (-, not observed; A-F according to Agerer 1987–2002). MT: mantle in plan view (-, not tested; A-Q refers to the types in Agerer 1987–2002). Cy: cystidia (-, not observed; A-P according to Agerer 1987–2002). Lh: Laticiferous hypae (-, not observed; + present). An: anastomoses (-, not observed; O, open; S, closed by a simple septum: C, closed by a clamp, Agerer 2002). Hs: hyphal surface emanating from the mantle and/or rhizomorphs (-, not observed; S, smooth; R, rough; C, crystals). Cl: clamps (+, clamps presence; -, not observed). Co: colour (WT, white; YE, yellow; OR, orange; GR, green; BL, blue; VI, violet; RD, red; BR, brown; D’BR, dark brown, BK, black). Sh: shape of mycorrhiza (Unr, unramified; Dic, dichotomous; Mon-pyr, monopodial-pinnate; Mon-pir, monopodial pyramidal; Irr-pin, irregularly-pinnate; Cor, coralloid; Tub, tuberculate, according to Agerer 1987–2002). ET: exploration type (C, contact; SD, short-distance; MF, medium distance fringe; MS, medium distance smooth; LD, long distance; PB, pick a back. Sensu Agerer, 2001). %ORAN: percentage of overall relative abundance based on total number of EcM root tips counted in 18 soil cores. ^a^Percentage value less than 1 (e-3).

### 3.2. Relative abundance descriptors: root tip number versus DW

**Figure 4.**
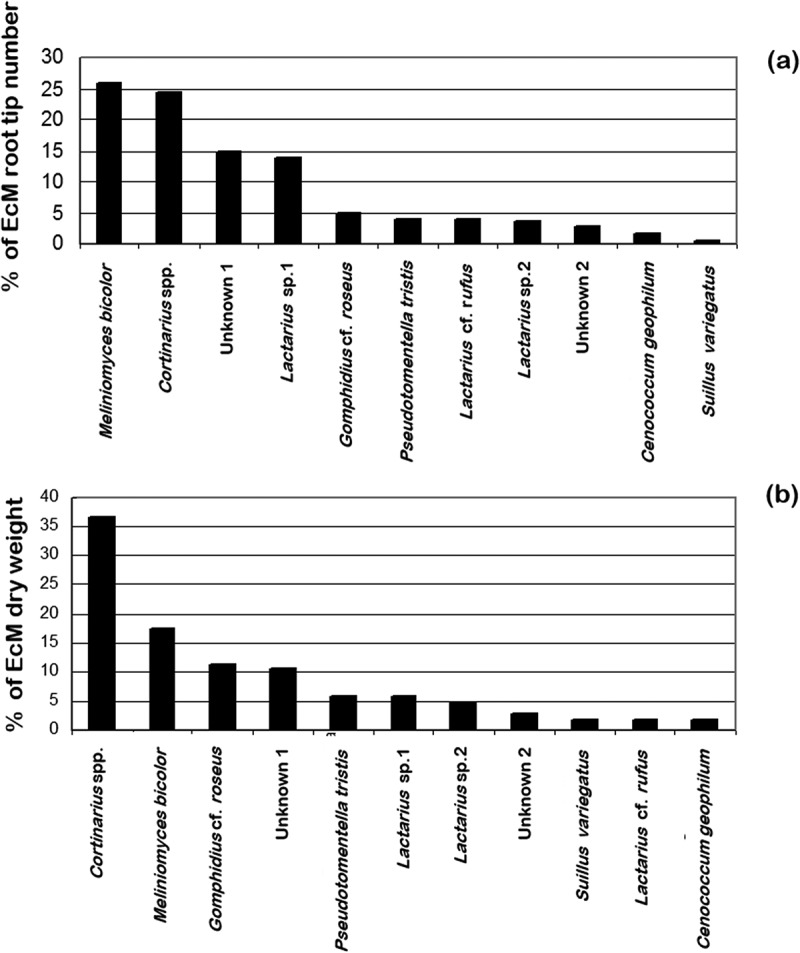
Comparative analysis of the per cent of relative abundance of 11 EcM fungal taxa along the transect. (a) Community structure based on per cent of EcM root tips number (*n* = 6689). (b) Community structure based on per cent of EcM dry weight (*n* = 992.6 mg).

**Figure 5.**
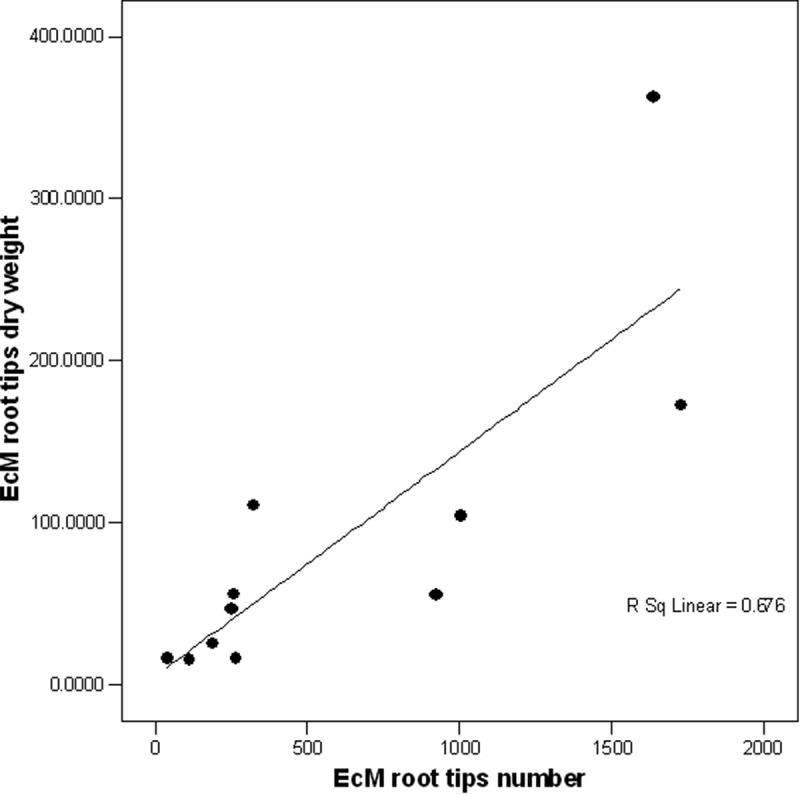
Relationship between number of EcM root tips (n = 6689) and dry weight (n = 992.6 mg). Result of regression analysis: r^2^ = 0.676, p < 0.01.

EcM relative abundance expressed as root tip number or DW gave a different picture of fungal community structure along the transect in the Scots pine stand changing the distribution of the EcM taxa, Figure 4 a-b. When expressed by root tips number, the predominant taxa was *M. bicolor* accounting with 26% followed by *Cortinarius* spp. with 24%. However, when the relative abundance was expressed in DW, a switch on dominance was observed with *Cortinarius* spp. as the most abundant with 37% followed by *M. bicolor* with 17%. When the number of EcM root tips was plotted against the DW for each group, there was a positive relationship (*r^2^ *= 0.676, *p* < 0.01); however, only 68% of the relationship was explained, Figure 5.

**Figure 6.**
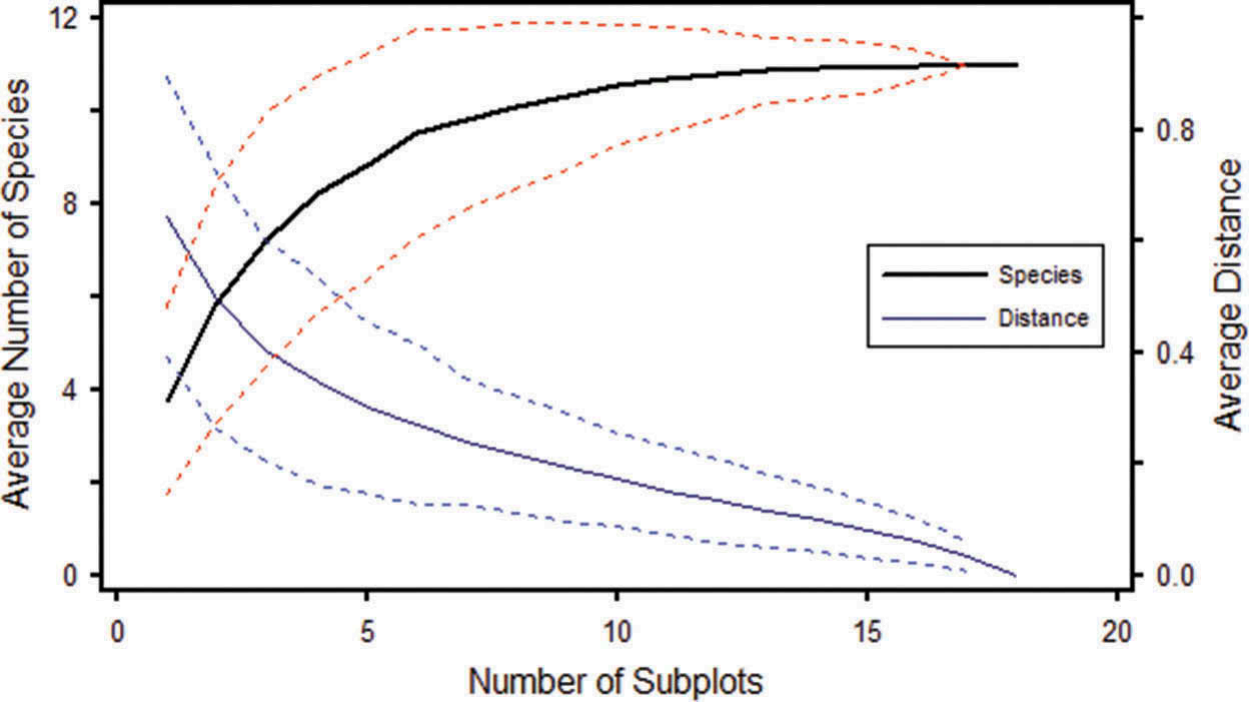
Species effort curve (heavy line) of 11 taxa recognised from 18 soil cores taken along a 125 m transect. The average of Sørensen (Bray-Curtis) distance between the subsamples is represented by the distance curve (light line). Dotted lines represent ± standard deviation.

The fungal species effort curve shows that 15 to 18 soil cores (diameter 4.5 cm; depth 12 cm) are required to collect all the EcM fungal taxa in the Scots pine stand sampled. First jackknife estimation indicates that no increase in the number of EcM taxa is expected by increasing the number of soil cores in this particular 50-year-old pine woodland, Figure 6.

### 3.3. Relationship between EcM taxa dissimilarity and sample points

**Figure 7.**
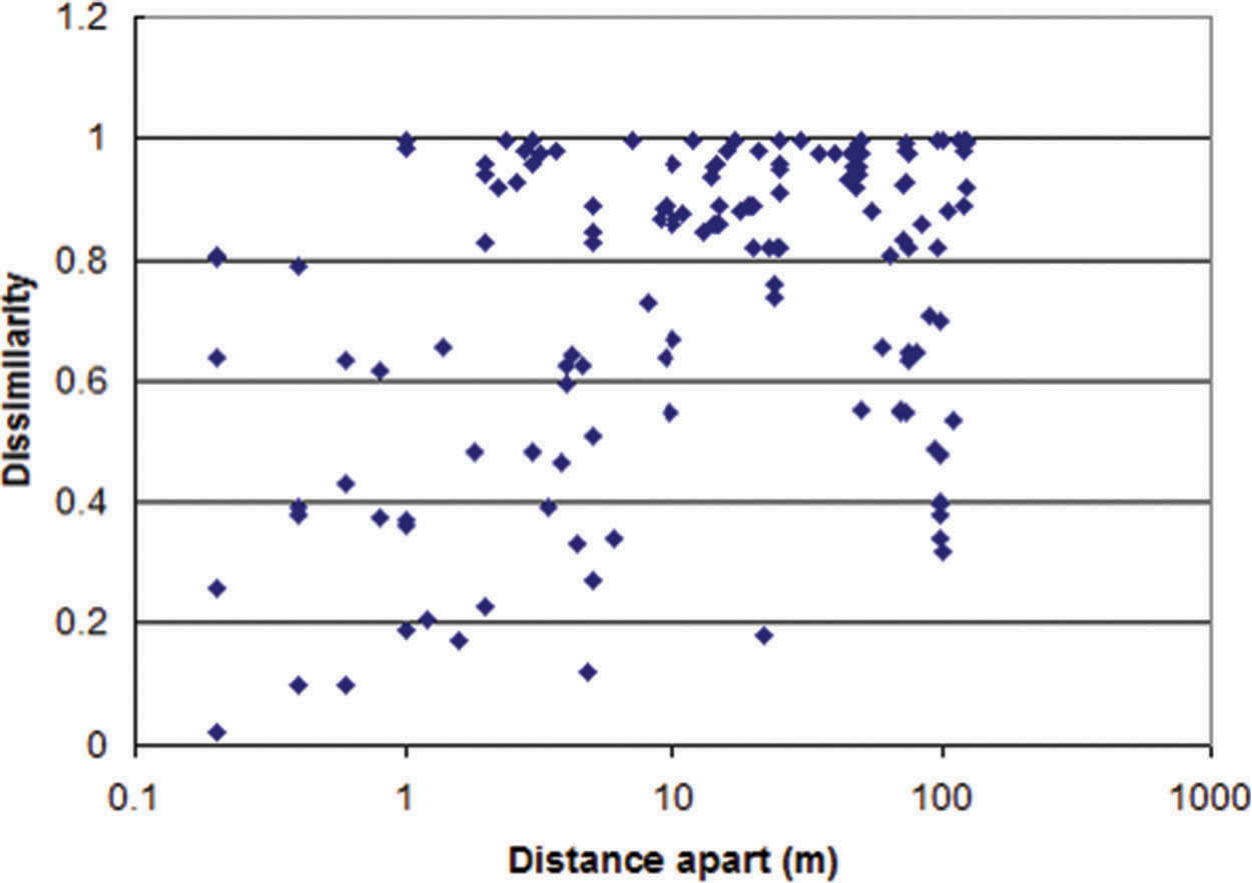
Relationship between taxa dissimilarity of 18 soil cores taken at set distances along a 125 m transect using Sørensen (Bray & Curtis) distance.

The Sørensen distance between each pair of plots was then plotted against the physical distance apart of that pair of plots along the transect, Figure 7. A highly significant relationship (*r^2^* = 0.16, *p* < 0.001) was found between Sørensen dissimilarity in soil cores and distance apart.

## 4. Discussion

### 4.1. EcM taxa detection

In this study, we confirm that the divide of EcM types into morphological groups based on gross morphology (morphotyping), and testing the hypothesis by anatomical characterisation (anatotyping) when discrepancies were observed is of great importance for the recognition and identification of EcM taxa in ecological studies, in line with Agerer (2002). Although this was a time-consuming exercise, it proved crucial to the detection of mixed taxa, e.g. *Piceirhiza bicolorata*-like EcM formed by *Meliniomyces bicolor* are frequently misidentified with *Cenococcum geophilum* EcM confirming Vrålstad et al.’s (2002) findings. In the case of the genus *Cortinarius* with “white” EcM (and considering that this genus is diverse in the study stand), we performed “identification probes” *sensu* Agerer (1991) tracing sporome-EcM, revealing that different taxa of *Cortinarius* frequently shared morpho-anatomical attributes which is in line with Rosling et al.’s (2003) findings. As a result, in the current study no further identification attempts were made from “blind probes” *sensu* Agerer (1991), grouping similar white EcM types in the taxon *Cortinarius* spp. for practical reasons. In some cases, distinctive sclerotia (≥0.5 to ≤1.0 mm) of *C. geophilum* (Figure 3. 10(a)) were present in soil cores 10 and 11 where the EcM morphotype was absent. Because is well known that *C. geophilum* lacks an apparent sexual stage (Horton and Bruns 2001) and that under environmental stress forms abundant sclerotia (Massicotte et al. 1992), this resting structure could be considered as an indicator of fungal presence in future ecological studies.

### 4.2. Why EcM fungal community change with descriptors?

The disparity found between the number and DW of EcM morpho-anatotypes can be explained in the general morphology of each mycobiont on pine roots, particularly the amount of extramatrical hyphae (explotation types *sensu* Agerer 2001). In the case of *Meliniomyces bicolor* (Figure 3.1) versus *Cortinarius* spp., Figure 3.2, the short emanating hypha arising from the mantle (*short distance* exploration type, Agerer 2001) in the former and the dense emanating elements attached to organic debris in the latter (*medium distance fringe* exploration type, Agerer 2001) made a clear distinction. Both taxa had almost the same level of abundance as number of root tips; however, *Cortinarius* spp. have twice the biomass than *M. bicolor*. Another study case is the tuberculate ectomycorrhiza of *Suillus variegatus* (Swartz ex Fr.) Kuntze with *long distance* exploration type (Agerer 2001), which increases in relative abundance when expressed by weight. These tubercules may contain 10 to 50 root tips (mean 32.75 ± 19.10 SD, *n *= 5) covered by a thick peridium-like layer. Because the removal of this peridium-like layer is needed to count the number of root tips (which is a time-consuming task), in future studies the tuberculate ectomycorrhizas of *Suillus* spp. will be considered as a unit according to their putative storage functional importance as discussed by Smith and Read (1997) and scored as one EcM root tip for practical reasons.

### 4.3. EcM community sampling effort and distribution

Ectomycorrhizal fungi are complex communities contrasting with the relatively simple host plant communities in boreal forests (Erland and Taylor 2002). High species richness, patchiness and their apparent non-random distribution are attributes of EcM fungal communities to be considered when developing reliable sampling protocols in ecological studies (Horton and Bruns 2001; Taylor 2002). In this study sampling, 18 soil cores (diameter 4.5 cm; depth 12 cm) along a transect is enough to collect most of EcM taxa occurring in the top 12 cm soil layer in this 50-year-old (stem exclusion: thicket phase) planted *Pinus sylvestris* stand. Testing sampling effort and EcM species detection in a 50-year-old Scots pine forest, Taylor (2002) report that *Cortinarius* was the most abundant genus in sporocarp abundance (42.3%) and species richness (25) but poorly represented (1.6%) as below-ground EcM which contrast with our findings where *Cortinarius* spp. was well represented below-ground in number and total biomass DW. Genney et al.’s (2006) study on a UK Scots pine stand showed that some species like *Cadophora finlandia*= *Meliniomyces bicolor* was distributed evenly with soil depth while the *Cortinarius* spp. EcM was concentrated in the upper 10 cm which is in line with the present study findings. Methods of scoring ectomycorrhizas by number of root tips versus DW provide a different picture of species composition; however, we found a relationship between the two measures and we can conclude that the number of EcM root tips is more suitable and less time consuming if we combine with a meticulous morpho-anatotyping study. The use of transects across gradients are helpful to describe maximum variation over short distances in a short period of time (Kent and Coker 1999; Toljander et al. 2006). However, in the current study the spatial arrangement of samples indicated that over short distances the dissimilarity was lower in contrast with the longer distances, suggesting that a random sampling across a wider plot could be more suitable in Scots pine woodlands.
