# Erratum

**DOI:** 10.1080/21501203.2017.1407529

**Published:** 2018-05-10

Villarreal-Ruiz, L, Neri-Luna, C. 2017. Testing sampling effort and relative abundance descriptors of belowground ectomycorrhizal fungi in a UK planted scots pine woodland. Mycology. https://doi.org/10.1080/21501203.2017.1394393

When the above article was first published online, the following references were omitted by mistake:

- Agerer R, Ammirati J, Blanz P, Courtecuisse R, Desjardin DE, Gams W, Hallenberg N, Halling R, Hawksworth DL, Horak E, Korf RP, Mueller GM, Oberwinkler F, Rambold, G, Summerbell RC, Tribel D and Watling R. 2000. Open letter to the scientific community of mycologists. Canadian Journal of Botany. 78:981–983
- Agerer R. 1991. Characterization of ectomycorrhiza. In: Norris JR, Read DJ, Varma AK, editors. Techniques for the study of mycorrhiza. Methods in microbiology. 23:25–73.
- Palmer MW. 1991. Estimating species richness: the second order jackknife reconsidered. Ecology. 72:1512–1513.
- Erland S and Taylor AFS. 2002. Diversity of ecto-mycorrhizal fungal communities in relation to the abiotic environment. In: van der Heiden MGA and Sanders IR, editors. Mycorrhizal ecology. Ecological Studies. Berlin: Springer; p. 163–200.

The caption for Figure 2 incorrectly read:

“(a) Illustration of transect line (blue dashed line) and replicated sampling points (red dots). (b) Field sampling procedure in stand 8. (1) transect line, (2) soil samples in a bucket, (3) soil corer. (c) Detail of soil sample (1) in the soil corer (2) (3) Ruler 15 cm.’’

This has now been corrected to:

“(a) Illustration of transect line (dashed line) and replicated sampling points (dots). (b) Field sampling procedure in stand 8. (1) transect line, (2) soil samples in a bucket, (3) split soil core sampler. (c) Detail of soil sample (1) in the split soil core sampler (2) (3) Ruler 15 cm.’’

The caption for figure 5 incorrectly read:

“Result of regression analysis: r2 = 0.676**, p < 0.01**.’’

This has now been corrected to:

“Result of regression analysis: r2 = 0.676, p < 0.01.’’

In Table 1 the reference to Agerer, mistakenly listed the year as 2006 in three places, this has now been corrected to Agerer 1987–2002.

These items have now been corrected in both the print and online versions of the journal.

Taylor and Francis apologises for these errors.

